# Giant Sigmoid Fecaloma Causing Complete Large Bowel Obstruction in a Patient With Intellectual Disability: A Case Report

**DOI:** 10.7759/cureus.107890

**Published:** 2026-04-28

**Authors:** Hugo E Mora Moreno, Noel Garcia Gonzalez, Alondra Y Ramirez Gomez, Javier Querea Vázquez

**Affiliations:** 1 General Surgery, Hospital General Dr. Miguel Silva, Morelia, MEX

**Keywords:** fecaloma, hartmann’s procedure, intestinal obstruction, large bowel obstruction, sigmoid colon

## Abstract

Fecaloma is a rare but clinically significant cause of large bowel obstruction, often associated with chronic constipation and underlying conditions affecting bowel motility. We report the case of a 50-year-old man with a history of intellectual disability who presented with a 10-day history of progressive abdominal pain, obstipation, abdominal distension, vomiting, and intolerance to oral intake. Physical examination revealed a distended abdomen with decreased bowel sounds and no signs of peritoneal irritation. Laboratory findings were unremarkable except for a mild elevation of lactate dehydrogenase (LDH). Contrast-enhanced computed tomography (CT) demonstrated marked colonic dilation with a heterogeneous intraluminal mass in the sigmoid colon, consistent with a fecaloma, causing complete large bowel obstruction without evidence of perforation or ischemia. Given the severity of the presentation and clinical deterioration, the patient underwent urgent exploratory laparotomy, confirming a large impacted fecaloma with significant proximal colonic dilation. A sigmoidectomy with end colostomy (Hartmann’s procedure) was performed without complications. The postoperative course was favorable, with adequate stoma function and no evidence of infection or leakage. Histopathological analysis confirmed chronic mechanical obstruction with adaptive muscular hypertrophy and pressure-related mucosal changes, without malignancy. This case highlights the importance of considering fecaloma as a differential diagnosis in large bowel obstruction, particularly in vulnerable populations. In addition, it underscores the role of timely imaging and surgical intervention in preventing severe complications.

## Introduction

Fecaloma is a rare clinical entity characterized by the accumulation of hardened fecal material forming a distinct intraluminal mass that cannot be expelled spontaneously. It most commonly occurs in the rectosigmoid region due to the narrower lumen and increased water absorption in the distal colon, which favors fecal desiccation and compaction [1]. Although frequently associated with chronic constipation, fecalomas have also been described in patients with neuropsychiatric disorders, reduced mobility, and underlying motility disturbances [2].

Intestinal obstruction remains a common surgical emergency, with large bowel obstruction accounting for a significant proportion of cases. The most frequent etiologies include malignancy, volvulus, and diverticular disease; however, fecaloma represents an uncommon and often underrecognized cause of mechanical obstruction [3]. In such cases, the clinical presentation may mimic more prevalent conditions, leading to potential delays in diagnosis and management.

Computed tomography (CT) plays a fundamental role in the evaluation of intestinal obstruction, as it allows accurate identification of the level, cause, and associated complications. It provides detailed information regarding bowel dilation, vascular status, and possible underlying etiologies, such as tumors or hernias, thereby guiding timely clinical and surgical decision-making [4].

Management strategies depend on the severity of obstruction and the patient’s clinical status. Although conservative approaches, such as enemas, laxatives, and endoscopic removal, may be effective in selected cases, surgical intervention is required in patients presenting with complete obstruction, failed conservative management, or signs of clinical deterioration [5].

We present the case of a middle-aged man with intellectual disability who developed a complete large bowel obstruction secondary to a giant fecaloma in the sigmoid colon, requiring urgent surgical management. This case highlights the importance of considering fecaloma as a differential diagnosis in patients with bowel obstruction, particularly in vulnerable populations, as well as the need for timely surgical intervention in advanced presentations.

## Case presentation

A 50-year-old man with a history of moderate intellectual disability, dependent on caregivers for daily activities, presented to the emergency department with a 10-day history of progressive abdominal pain, absence of bowel movements, and failure to pass flatus. According to his caregivers, he had a history of chronic constipation, characterized by fewer than three bowel movements per week and frequent use of laxatives.

The abdominal pain had an insidious onset, was continuous, diffuse, and progressively increasing in intensity. It was associated with nausea, multiple episodes of vomiting, abdominal distension, and intolerance to oral intake. Due to the patient’s underlying intellectual disability and limited ability to communicate symptoms, the clinical history was restricted and primarily obtained from family members. On admission, the patient exhibited clinical signs of dehydration.

Laboratory findings are summarized in Table 1. An elevated lactate dehydrogenase (LDH) level was noted, without other clinically significant abnormalities, suggesting the absence of intestinal ischemia at the time of initial evaluation. 

**Table 1:** Laboratory parameters at admission. Admission laboratory results showing normal hematologic and inflammatory markers, with mild LDH and transaminase elevations, consistent with a localized colonic obstruction without systemic inflammation.

Parameter	Result	Reference range
White blood cell (WBC) count	7.4 × 10³/µL	4.5-10.0 × 10³/µL
Hemoglobin (Hb)	15.3 g/dL	13.5-17.5 g/dL
Platelet count	235 × 10³/µL	150-400 × 10³/µL
Lactate dehydrogenase (LDH)	449 U/L	120-300 U/L
C-reactive protein (CRP)	1.2 mg/L	0-6 mg/L
Aspartate aminotransferase (AST)	42 U/L	0-40 U/L
Alanine aminotransferase (ALT)	41 U/L	0-41 U/L
Glucose	87 mg/dL	74-109 mg/dL

**Figure 1:**
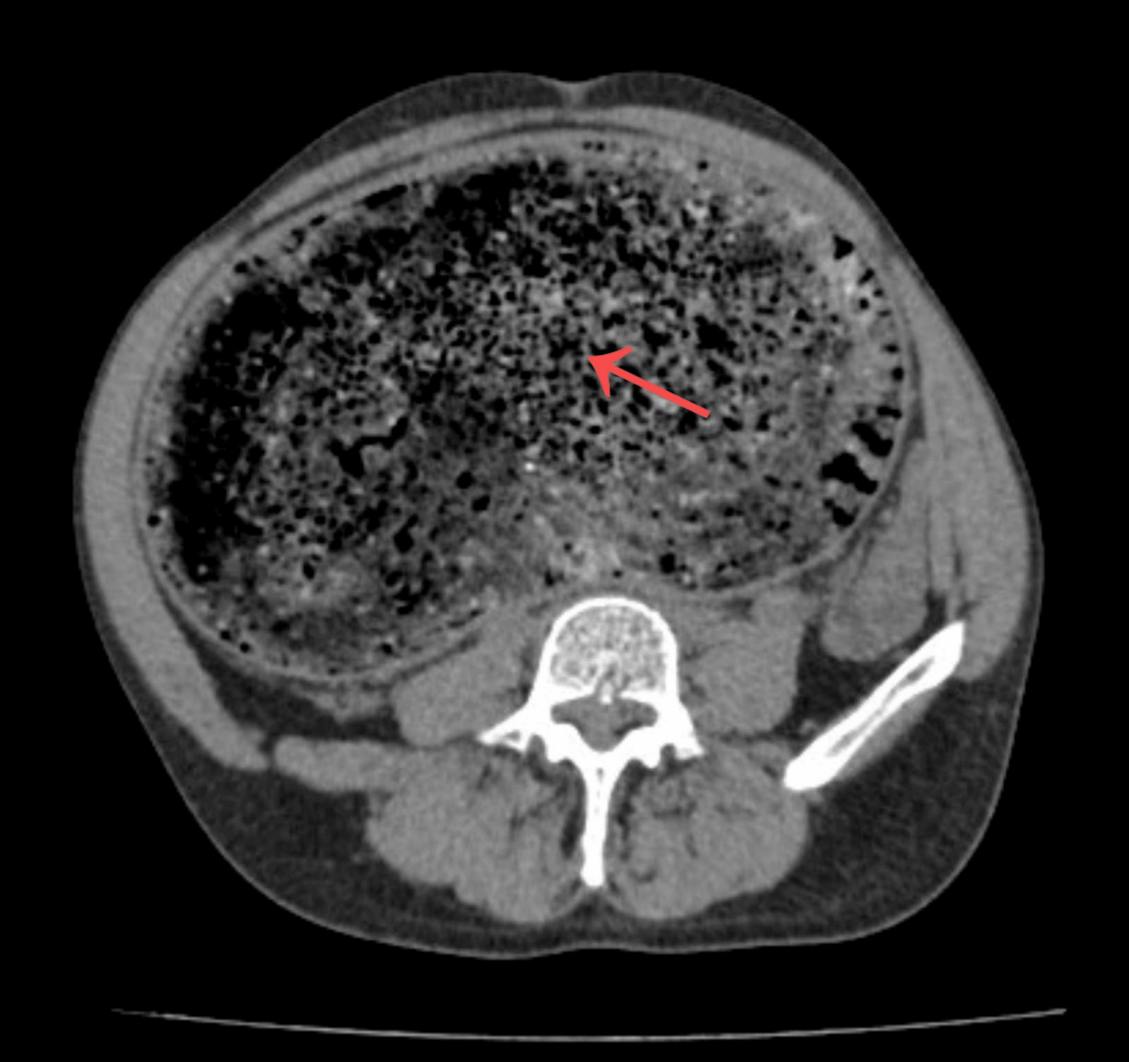
Axial CT demonstrating sigmoid fecaloma. Axial contrast-enhanced computed tomography (CT) image showing marked colonic dilation with a well-defined, heterogeneous intraluminal mass with a mottled gas pattern in the sigmoid colon (arrow), consistent with fecaloma causing distal large bowel obstruction.

**Figure 2:**
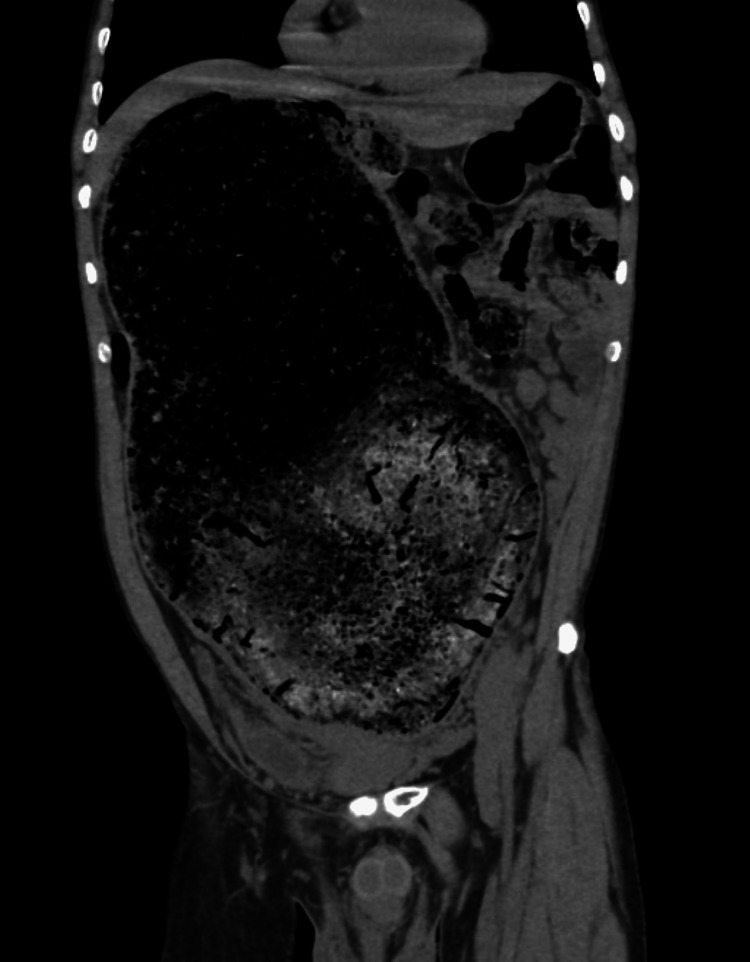
Coronal CT demonstrating sigmoid fecaloma. Coronal CT reconstruction demonstrating significant dilation of the proximal colon upstream from an obstructing fecaloma measuring 40 cm in length and 25 cm in width, without evidence of free air or perforation. CT: computed tomography

**Figure 3:**
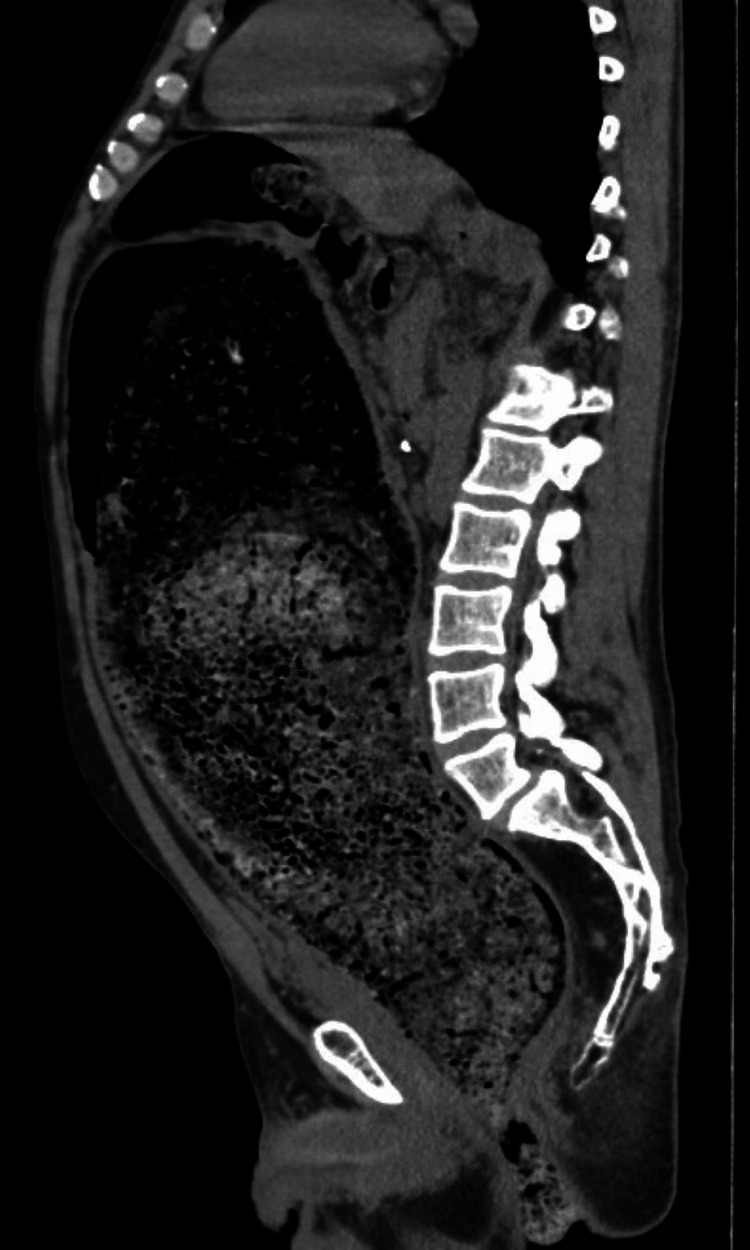
Sagittal CT illustrating the extent of obstruction. Sagittal CT reconstruction depicting the full extent of colonic dilation and the impacted fecaloma at the sigmoid level. CT: computed tomography

An abdominal ultrasound was initially performed, showing marked colonic distension with abundant intraluminal echogenic material, suggestive of fecal loading. Subsequently, a contrast-enhanced CT scan of the abdomen and pelvis demonstrated significant colonic dilation, predominantly at the level of the sigmoid colon, with heterogeneous mottled intraluminal content consistent with a fecaloma. This finding was indicative of a complete large bowel obstruction, with marked dilation of the proximal colon. No evidence of perforation, pneumoperitoneum, or radiological signs of intestinal ischemia was identified (Figures 1-3). No plain abdominal radiography was performed before CT imaging. 

Given the diagnosis of complete large bowel obstruction and the patient’s clinical deterioration, urgent surgical management was undertaken due to the high risk of progression to ischemic or perforative complications.

**Figure 4:**
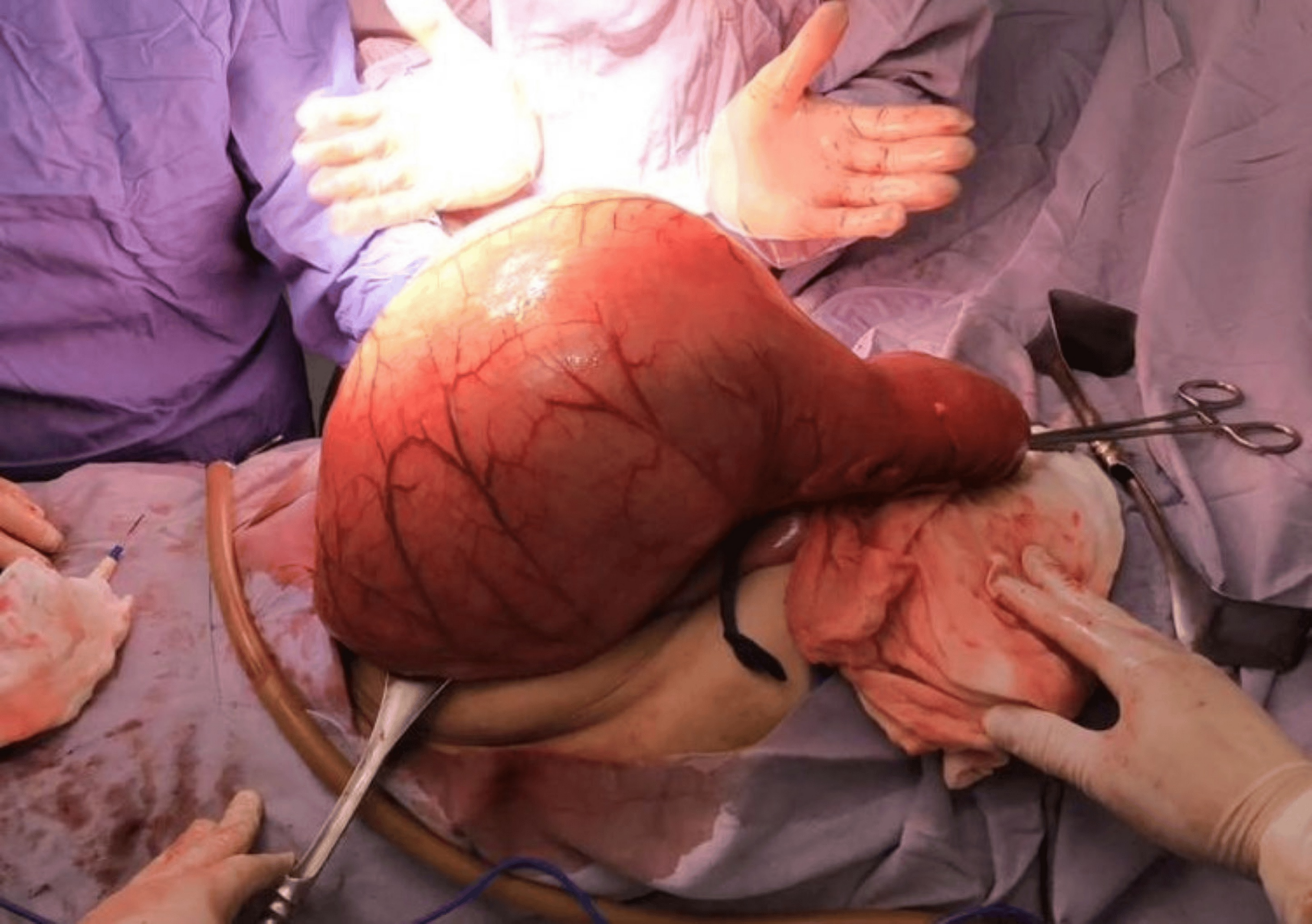
Intraoperative view of the resected segment. The resected segment shows marked proximal colonic dilation, consistent with mechanical obstruction secondary to fecaloma.

Under general anesthesia, an exploratory laparotomy was performed, revealing a large fecaloma impacted in the sigmoid colon, associated with significant proximal colonic dilation (Figure 4).

**Figure 5:**
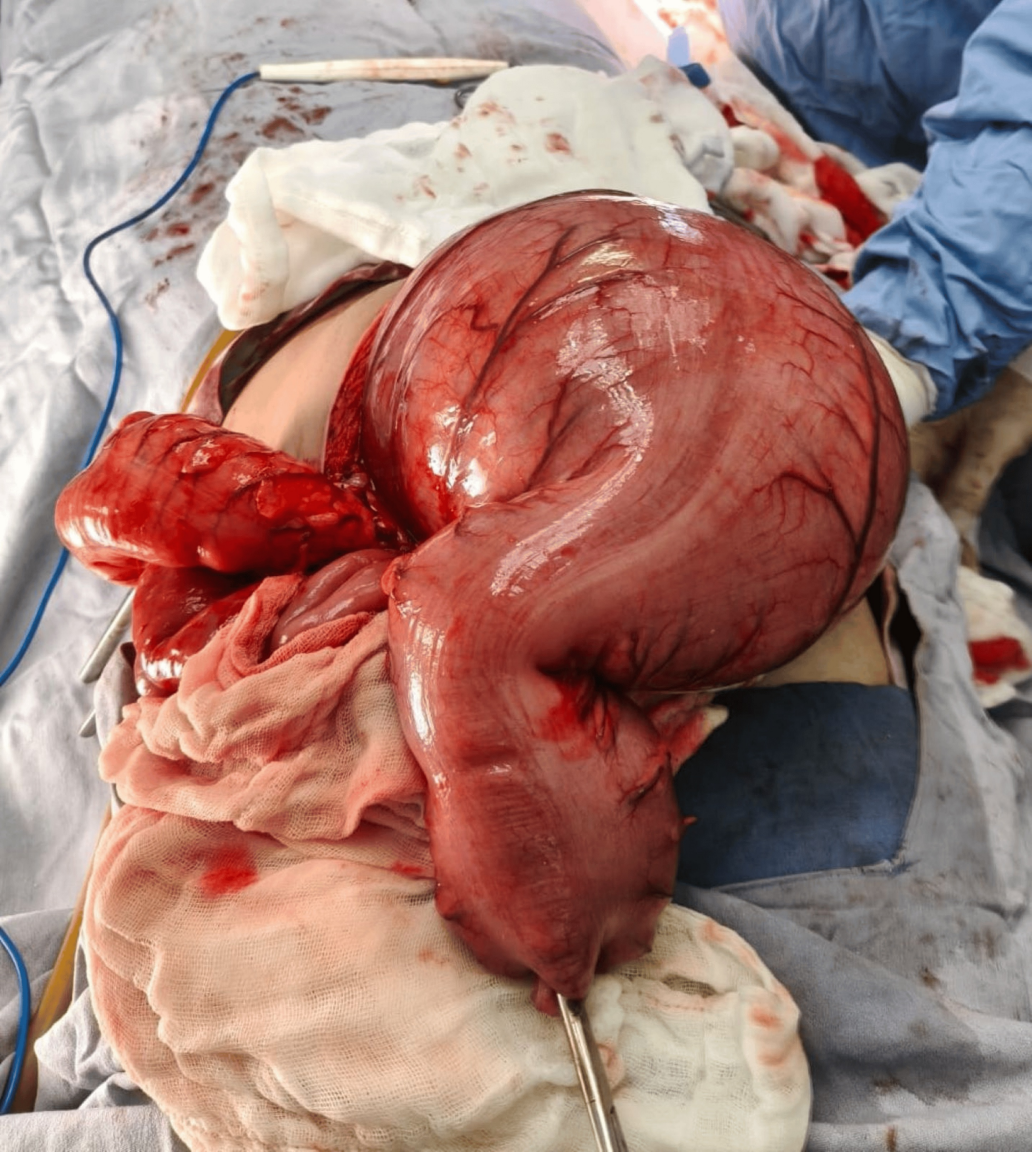
Surgical specimen with impacted fecaloma. Resected sigmoid colon specimen showing significant luminal distension and a large impacted fecaloma responsible for the obstruction.

**Figure 6:**
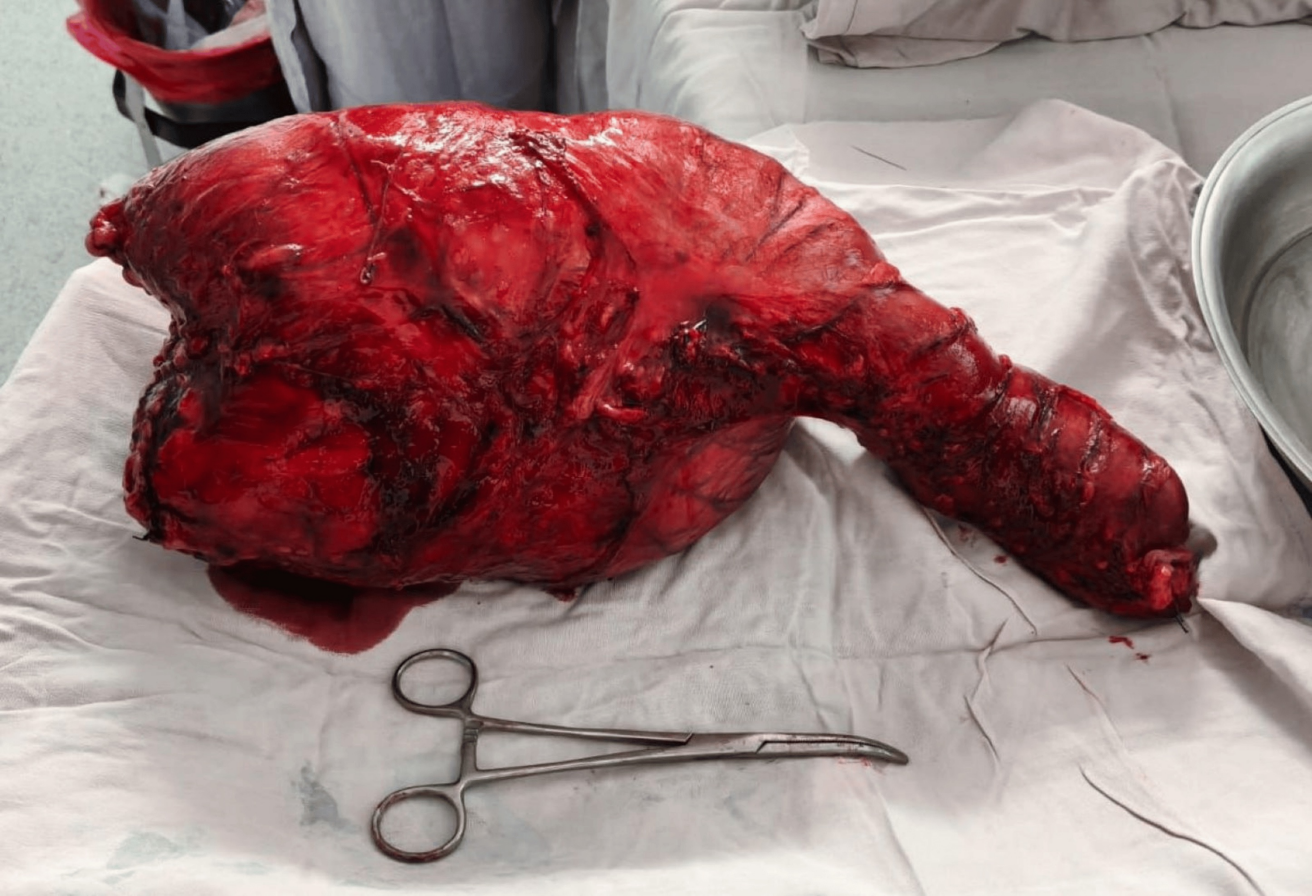
Resected segment. Gross examination of the resected colonic segment revealing a large fecaloma occupying the lumen, confirming the cause of obstruction. All images are original and provided by the authors.

An emergency sigmoidectomy was performed due to complete large bowel obstruction and marked colonic distension. Given the severe dilation of the proximal colon, the risk of anastomotic failure, and the patient’s clinical condition, Hartmann’s procedure was deemed the safest surgical option. The rectal stump was closed at a low level due to the distal extent of the fecaloma and significant involvement of the sigmoid colon, precluding a more proximal transection. An end colostomy of the descending colon was created. The resected specimen demonstrated a markedly dilated sigmoid colon containing a large impacted fecaloma, confirming the mechanical cause of obstruction (Figures 5-6).

During the postoperative period, the patient showed favorable clinical evolution under close medical supervision, with adequate tolerance to enteral feeding and functional fecal output through the stoma. The intra-abdominal drain showed no evidence of intestinal leakage or signs of intra-abdominal infection and was removed in a timely manner. The patient was discharged due to clinical improvement with outpatient follow-up.

At follow-up, the patient was in good general condition, with a functional stoma, a clean surgical wound with appropriate healing, and a soft, non-tender abdomen without signs of peritoneal irritation. Histopathological examination revealed chronic mechanical obstruction secondary to fecaloma, with adaptive changes in the intestinal wall, including muscular hypertrophy, as well as pressure-related changes characterized by mucosal flattening and chronic inflammatory infiltrate, with no evidence of malignancy. The resected specimen measured 39 × 24 × 18 cm. Sutures were removed without complications. A plan for elective stoma reversal has been considered, contingent upon the patient’s clinical status and adequate preoperative evaluation, including assessment of the distal rectal stump.

## Discussion

Fecaloma represents an uncommon but clinically significant cause of large bowel obstruction, often associated with chronic constipation and underlying neuropsychiatric or neurological conditions that impair colonic motility [6]. In the present case, the patient’s intellectual disability likely contributed to delayed symptom recognition and chronic bowel dysfunction, predisposing to progressive fecal accumulation and eventual obstruction.

Although large bowel obstruction is frequently attributed to malignancy, volvulus, or diverticular disease, fecaloma accounts for a rare etiology and may be underdiagnosed due to its nonspecific clinical presentation [7]. Patients typically present with abdominal pain, distension, constipation, and vomiting, as observed in our case, which can mimic more common obstructive pathologies and delay appropriate management.

CT plays a pivotal role in diagnosis, allowing differentiation between fecaloma and other causes of obstruction. Characteristic findings include a well-defined intraluminal mass with a mottled gas pattern and associated proximal bowel dilation [8]. In our patient, CT imaging was essential not only for confirming the diagnosis but also for excluding complications, such as perforation or ischemia, which significantly influence therapeutic decision-making.

Management of sigmoid fecaloma is not standardized and largely depends on the clinical presentation, size of the fecal mass, and the presence of complications. Current evidence supports a stepwise approach, beginning with conservative measures, such as laxatives, enemas, and manual disimpaction. However, these strategies may be insufficient in cases with large or long-standing fecalomas, similar to what was observed in our patient [9,10].

In this context, several reports emphasize the growing role of endoscopic management, which allows both diagnosis and treatment through direct fragmentation and removal of the fecaloma. Compared to conservative therapy, colonoscopic intervention appears to offer higher success rates in refractory cases while avoiding the morbidity associated with surgical procedures [9-11]. In our case, the persistence of symptoms despite initial conservative management, including rectal enema, highlights the limitations of non-operative treatment and supports the need for timely escalation.

Nevertheless, endoscopic treatment may not be feasible in all patients, particularly in the presence of severe obstruction, significant bowel wall compromise, or failed previous attempts. In such scenarios, surgical intervention remains the definitive option, albeit associated with increased risk [10,11]. Overall, our findings are consistent with the current literature and reinforce the importance of an individualized, stepwise approach. This case underscores the value of early recognition and appropriate therapeutic escalation, as well as the role of endoscopic management as an effective intermediate strategy before considering surgery.

In the present case, the patient exhibited signs of complete large bowel obstruction with progressive clinical deterioration, warranting urgent surgical management. The decision to perform Hartmann’s procedure was based on several factors, including significant colonic dilation, risk of perforation, and the patient’s overall clinical status. This approach is widely supported in emergency settings where primary anastomosis carries a higher risk of complications [12].

The histopathological findings in our case demonstrated features consistent with chronic mechanical obstruction, including adaptive muscular hypertrophy and pressure-induced mucosal changes, reflecting prolonged fecal stasis and increased intraluminal pressure. These findings support the chronic nature of the condition and highlight the importance of early diagnosis to prevent irreversible structural damage [11].

Sigmoid fecal impaction may progress to serious complications, most notably stercoral perforation secondary to pressure-induced necrosis of the colonic wall, leading to localized ischemia and eventual rupture. Previous reports of sigmoid fecaloma have also described pressure-related mucosal ulceration and ischemic changes, which may advance to transmural necrosis as a consequence of sustained intraluminal pressure and persistent fecal stasis [11]. 

Furthermore, this case emphasizes the need for increased clinical awareness of fecaloma as a differential diagnosis in patients presenting with bowel obstruction, particularly in high-risk populations such as those with cognitive impairment or limited access to healthcare [13]. Early diagnosis and timely intervention are essential to reduce morbidity and improve outcomes.

## Conclusions

Fecaloma is an uncommon but important cause of large bowel obstruction, particularly in patients with risk factors such as cognitive impairment and chronic constipation. CT is the key to diagnosis and assessment of complications. Although conservative management may be attempted, complete obstruction requires timely surgical intervention to prevent serious complications.
